# Poor-Law Infirmaries: Their Possibilities in the Furtherance of Medical Education

**Published:** 1907-04-13

**Authors:** T. R. St. Johnston

**Affiliations:** Senior Assistant Medical Officer, Lewisham Infirmary, London, S.E.


### POOR-LAW INFIRMARIES: THEIR POSSIBILITIES IN THE FURTHERANCE OF MEDICAL EDUCATION.

By T. R. St. JOHNSTON, M.R.C.S., L.R.C.P., M.S.A., Senior Assistant Medical Officer, Lewisham Infirmary, London, S.E.

"Particularly he entertained me with discourse of an infirmary, which I mightily approve of; and will endeavour to promote it, being a worthy thing, and of use, and will save money."—Pepys' "Diary," January 29, 1666.

### I. A HUGE WASTE OF GOOD MATERIAL.

What is the main object of a Poor-law infirmary? At first glance one would say, "To take care of the infirm poor." Though this is the fundamental principle on which these institutions are founded, there are wider aspects to the question. Perhaps the summing up of the quaint old diarist, as written above, yields the clearest answer, "A worthy thing, and of use, and will save money." It is true that the infirmary of which he wrote was to be founded for sick and wounded seamen, but the same claims may be equally applied to any modern Poor-law infirmary.

That the relief of suffering and pain is a worthy thing no one will deny, that to collect the infirm poor and administer to their wants in one central building instead of at their own homes saves money is also a proved fact; but do we make the most *use* of our infirmaries? The question of infirmaries touches very closely a great social problem, namely, how far the successful ones in life ought to contribute to the maintenance of the unsuccessful. It is a noble idea that the more prosperous should help the unfortunate, but it is also a business transaction. And if—in the form of rates and taxes—one portion of the people is compelled to provide for the other portion, what return are the contributors to have for their money? That the system tends to a happy and peaceful community is true, that it tends to the curtailment of disease is also true; but cannot some further advantage be reaped from it? In large factories and commercial enterprises the actual articles produced often only just pay the cost of production, and the biggest profits are made from what the casual observer would call "waste materials." In the same way the management of communities of people—in other words, cities—must be looked upon as a big commercial enterprise, and from a multitude of little waste products is the profit, or the return to the ratepayer, to be made. One of these by-products, at present but little used, is the opportunity for the advancement of medical education—and so indirectly for the promotion of the better health of the citizens—that exists in our infirmaries.

### The Medical Work of Infirmaries.

Most Union infirmaries, both in London and in the big provincial centres, contain from 250 to 800 beds, and are staffed by a resident superintendent and one or two assistant medical officers. It is difficult to speak collectively of all, for the class of cases they receive varies considerably with the geographical position of the Union. For instance, an infirmary within the four-mile radius of London, and close to a large hospital, will probably not get the accidents and acute work that will come to an outlying institution, which has to take on the duties of a general hospital in addition to its own. But even in the more central infirmaries there is a wealth of material in the shape of interesting clinical cases that is seldom realised by members of the profession who have not come into actual contact with them. One is too apt to think that the patients in an infirmary consist of two classes only—those that are incurable and have been transported from the hospitals merely to die, and those that are sent across from the workhouse with the simple diagnosis "Senectus" written on their cards. This is very erroneous. Take the average infirmary of 500 beds. There is, say, a resident superintendent, and he has two resident assistant officers. The superintendent may be either a man of administrative capabilities who confines himself almost entirely to that sphere (when he will quite earn his salary, for there is a vast amount of work in the administration of a large infirmary), or interested in his purely professional work and he may take a large share in the medical attendance on the patients. Occasionally, by dint of herculean labours, he may satisfactorily fulfil both duties of his post.

The two assistant officers usually divide the beds equally between them. Supposing there are 400 patients, there will thus be 200 patients to each assistant medical officer provided, as is usually the case that all the beds are occupied. Allowing the hours of morning work to be from 9.30 to 1.30 o'clock (and it is to be continuous work, with no rests in between), he will be able to devote to each patient under his care the lengthy time of one and one-fifth minute! And during his round he will probably have to dress a number of surgical cases, perform one or more minor operations, and undertake catheter, ophthalmoscopic and laryngoscopic work, not to speak of the administration of anæsthetics in conjunction with his colleagues. In fact, it is often far into the afternoon before he is able to finish even the routine work. How can he find time properly to go over his chest and nervous cases, or his gynæcological examinations? All the afternoon one of the assistant medical officers will have to be on duty, admitting and examining fresh cases, while the other gets an opportunity for fresh air and exercise. In the evening there is the night round to do, and casual admittances are coming up at intervals throughout the day. At night there are often obstetric cases, not infrequently "difficult labours," sent in by the parish doctor, for which the medical officers will have to be called up. With such a time-table as this the more scientific aspects of medical work such as bacteriological examinations, blood-counts, delicate urinary analyses, etc., can seldom be undertaken.

Here, then, is a harvest of clinical work, and reaped only by two or three medical men, and even by them, through pressure of time, not to its fullest advantage. When one finds there are no fewer than 18,000 *beds* in the infirmaries of London alone, one begins to realise what a large field for medical work is here waiting.

So far I have not yet mentioned the operative surgical work carried on in an infirmary, and about this also mistaken notions are current. I once heard a medical man say, "You surely don't have operating-theatres in an infirmary. I thought that all cases requiring operation had to go to the nearest hospital." Far from it. Practically all the infirmaries under the Local Government Board, especially those in London, are fitted up with first-class theatres, and with all the latest equipment necessary for carrying on the surgical work of the institution. Operations are usually performed by the superintendent, who is nearly always an experienced surgeon, and occasionally even takes a high place among the operating surgeons of the Metropolis. One of the assistant medical officers gives the anæsthetic and the other acts as surgical assistant. From the official books of one of the London infirmaries I find that during the last three months there have been over 150 operations performed; of which 23 were "abdominals," including two perforated gastric ulcers and an obturator hernia; seven were urinary cases, as lithotomies, urethrotomies, prostatectomies, etc.; one or two eye operations, seven mastoids, and a cephalotripsy—a record that could surely hold its own with many of the smaller non-teaching general hospitals.

### A Scheme to Utilise their Clinical Material.

But though there is all this surgical work to be seen, yet I do not think that surgery is the most valuable part of the infirmary work from the point of view of medical education. Surgical operations can, after all, be best seen and appreciated at the large teaching hospitals, where the greatest surgeons of the world make it their duty to instruct students in their art. Surgery is the essence of the hospitals, and to the major operations (and in medicine, the obscure diseases) the student gives his mind when he is reading for his "Final." How many students learn anything about *treatment* while still at the hospital? They see in the wards cases of this and that disease, and on the operating-table operations which are recorded in the journals for their rarity; but how many students when first qualified can treat a case of simple asthma or properly operate for tonsils and adenoids? These are the things they require as general practitioners, and it is to general practice that nine-tenths of them ultimately turn. Of course, if a student is lucky enough to get a house appointment after qualifying, so much the better for him; but students are many, and house appointments few. Can we, then, see in our Poor-law infirmaries a first glimpse of the axiom laid down by old Pepys, "A worthy thing, and of use, and will save money"? Can any scheme be made workable that will turn the clinical cases to be cases to be seen in the infirmaries to advantage as seen in the infirmaries to advantage as a means of medical training and education?

In the first place, there must be no revolutionary methods, for any scheme that may be evolved in a few minutes of thought and set in motion with perhaps not more consideration, is bound to fail in the end. It is only after a plan has been weighed and tried and deliberated upon from all points of view, and then gradually and slowly brought into being, that it can ever show its true value. Newly qualified students obviously cannot become superintendents or assistant medical officers, for even the assistant medical officers are practically in full charge of their patients, and have to have that experience which is necessary to give the self-reliance needed on critical occasions. When the superintendent is away there are no "honoraries" for them to fall back upon as a house-surgeon may do in a hospital. They have themselves been all through the routine of the various hospital appointments and are men who have settled down to the service of the Local Government Board. Moreover, a scheme is required whereby a considerable number of students may benefit by the practical teaching offered by an infirmary.

### Resident Clinical Assistantships.

Now, the Metropolitan Asylums Board have recently instituted at their fever hospitals an innovation which is of great value—namely, the appointment of resident clinical assistants for short periods of three months each. The authorities at these hospitals have officially recognised what has long been understood in general hospitals, that students can get but a superficial knowledge of their work by casually going round the wards for an hour or two each week, and that a real knowledge of the practical side of their profession can only be gained by a resident appointment. Could this arrangement be applied in any way to the Poor-law infirmaries?

Here there is a splendid field of clinical material, and of just the sort that is required for the best training of a medical man, the ordinary everyday ailments that occur in general practice. One might say, "Why not throw the infirmaries open straight away to student ward-classes, as carried on at the hospitals?" But this is too drastic a change, and would mean a complete upheaval of the present state of things, and a disorganisation which would hardly repay any possible benefits that might be obtained.

In the first place, if the infirmaries became ordinary teaching institutions, on exactly the same lines as the hospitals, would there be enough students to justify such an innovation? In a few schools, it is true, there are far too many students to give them a fair chance of bedside work; but for the most part there is usually enough clinical material in the hospitals for education as regards ward-classes and operations. What students require is *practical* knowledge—to be able to treat the patients at first-hand, and to know what to do in the various emergencies that continually confront a house-surgeon. And this knowledge they would be able to acquire if for a short time they could become resident clinical assistants.

